# Repellency, Fumigant Toxicity, Antifeedent and Residual Activities of *Coridothymus capitatus* and Its Main Component Carvacrol against Red Flour Beetle

**DOI:** 10.3390/molecules29174255

**Published:** 2024-09-08

**Authors:** Hassan M. Eltalawy, Huda El-Fayoumi, Shawky M. Aboelhadid, Saleh Al-Quraishy, Almahy M. El-Mallah, Fatma Tunali, Atalay Sokmen, Dimitra Daferera, Abdel-Azeem S. Abdel-Baki

**Affiliations:** 1 Zoology Department, Faculty of Science, Beni-Suef University, Beni-Suef 62511, Egypt; hassanmegahed729@gmail.com (H.M.E.); huda_fayoumi10@yahoo.com (H.E.-F.); elmahi.hussien@science.bsu.edu.eg (A.M.E.-M.); aabdelbaki@science.bsu.edu.eg (A.-A.S.A.-B.); 2 Parasitology Department, Faculty of Veterinary Medicine, Beni-Suef University, Beni-Suef 62511, Egypt; 3 Zoology Department, College of Science, King Saud University, Riyadh 12211, Saudi Arabia; squraishy@ksu.edu.sa; 4 Department of Pharmacy, Botanic Garden “Giardino dei Semplici”, “G. d’Annunzio” University, Via dei Vestini 31, 66100 Chieti, Italy; turali.fatma@gmail.com; 5 Department of Plant Production and Technologies, Faculty of Agriculture and Natural Sciences, Konya Food and Agriculture University, Konya 42080, Turkey; atalay.sokmen@gidatarim.edu.tr; 6 Laboratory of General Chemistry, Agricultural University of Athens, Iera Odos 75, 118 55 Athens, Greece; daferera@aua.gr

**Keywords:** *Tribolium castaneum*, *Coridothymus capitatus*, carvacrol, toxicity, repellency, feeding deterrent

## Abstract

*Tribolium castaneum* is a challenging pest of stored products, causing significant economic losses. The present study explored the efficacy of *Coridothymus capitatus* essential oil and its primary constituent, carvacrol, as eco-friendly alternatives for managing this pest. To evaluate their insecticidal potential, repellency, fumigant toxicity, and antifeedant properties, progeny inhibition assays were performed. Carvacrol exhibited superior repellency compared to the essential oil, achieving a 92% repellency rate at 2 mg/cm^2^. Both compounds demonstrated significant fumigant toxicity against *T. castaneum*, with LC_50_ values of 168.47 and 106.5 μL/L for the essential oil and carvacrol, respectively, after 24 h. Carvacrol also outperformed the essential oil in antifeedant activity, inducing an 80.7% feeding deterrence at 1.17 mg/g. Moreover, both treatments effectively suppressed the development of the pest’s progeny. These results collectively underscore the potent insecticidal properties of *C. capitatus* essential oil and carvacrol, particularly carvacrol, as promising candidates for the sustainable management of *T. castaneum* in stored product protection.

## 1. Introduction

The red flour beetle, *Tribolium castaneum* (Herbs), is considered one of the world’s most destructive pests for processed foods and stored products [1]. It has a wide range of target products, including cookies, beans, spices, pasta, cake mixes, dry pet food, dried flowers, chocolate, and almonds [2]. *Tribolium castaneum* is primarily found in tropical and subtropical regions and causes significant economic losses, posing a challenge to the value chain for grains [2,3]. Both the adults and larvae of this insect feed on broken and finely powdered grains, causing serious damage to stored seeds. These pests have a rapid population growth rate, which could lead to large economic losses if not properly managed [4]. The abdomen glands secrete benzoquinones and emit a large quantity of exuviae throughout their metamorphosis, resulting in unpleasant odors and colors in the products. As a result, it is classified as a pest that must be constantly monitored and managed [2,3].

The management of this pest in stored products has traditionally relied on chemical insecticides, particularly organophosphates, phosphine, and pyrethroids [5]. However, the excessive reliance on these synthetic compounds has resulted in a range of negative consequences. Environmental pollution, escalating treatment costs, and the development of pest resistance have become significant [6]. Moreover, the insect’s tolerance to multiple pesticides, including malathion and phosphine, has further complicated control efforts [7]. To overcome resistance, higher pesticide doses and more frequent applications are often employed, exacerbating environmental contamination, increasing economic burdens, and posing heightened risks to human health [8].

Thus, it is crucial to develop novel insecticides with reduced environmental and human health impacts [9,10]. Plant-derived compounds, characterized by their diverse biological activities, emerge as promising alternatives to synthetic insecticides [11,12]. A shift to plant-based products and their bioactive ingredients is consistent with sustainable development objectives. These botanical insecticides offer the advantages of being environmentally friendly, biodegradable, and are often effective against pest insects [13].

*Coridothymus capitatus*, also known by several synonyms such as *Thymus capitatus* and *Thymbra capitata*, is a plant native to Mediterranean Europe and Turkey. Commonly referred to as conehead thyme, Persian-hyssop, and Spanish oregano, it is widely recognized for its essential oils (EOs), which have proven effectiveness in pest control. The essential oils extracted from these plants are known for their potent antimicrobial, insecticidal, and acaricidal properties, making them valuable in natural pest management strategies [14]. The *Thymus* genus, to which *C. capitatus* belongs, is renowned for its diverse biological properties attributed to compounds like carvacrol, thymol, p-cymene, and γ-terpinene, known for their potent antibacterial activity [15]. Several studies have demonstrated the repellent and insecticidal effects of the members of the genus *Thymus* species against the red flour beetle, *Tribolium castaneum*. However, a comprehensive comparison of their toxicity, repellency, and fumigant properties remains scarce. Therefore, the main objective of the present study was to assess the insecticidal efficacy of *C. capitatus* extract and its major component, carvacrol, against *T. castaneum*.

## 2. Results

### 2.1. GC-MS of Coridiothymus capitatus Extract

**Figure 1:**
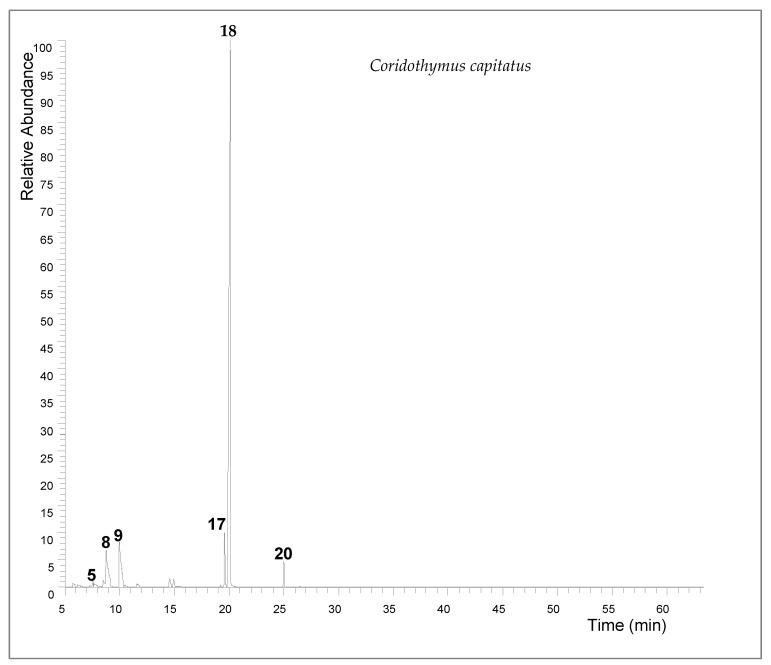
The total ion chromatogram (TIC) of *Coridothymus capitatus* essential oil (5 myrcene; 8 p-cymene; 9 *γ*-terpinene; 17 thymol; 18 carvacrol; 20 *β*-caryophyllene).

The extraction yield of *Coridothymus capitatus* essential oil was 2.7% (*v*/*w*). Gas chromatographic analysis revealed a complex composition, with carvacrol being the predominant constituent, making up 71.14% of the oil. Other significant components included γ-terpinene (8.3%), p-cymene (7.69%), and thymol (5.02%) (Table 1 and Figure 1).

### 2.2. Repellency Assay

The repellency against *T. castaneum* was found to be concentration-dependent for both *C. capitatus* essential oil and carvacrol. Carvacrol exhibited significantly greater repellency than *C. capitatus* extract (*p* < 0.001, F = 51.66), while both essential oils showed significantly higher repellency compared to the control (*p* < 0.001, F = 50.38). At the highest concentration (2 mg/cm^2^), DEET, carvacrol, and *C. capitatus* essential oil achieved repellency percentages of 100%, 92%, and 72%, respectively, after three hours. The concentration required to repel 50% of insects (RC_50_) was 0.42 mg/cm^2^ for carvacrol and 1.05 mg/cm^2^ for *C. capitatus* extract (Table 2).

### 2.3. Fumigant Toxicity Assay

Both *C. capitatus* essential oil (F = 312.62, *p* < 0.001) and carvacrol (F = 165.01, *p* < 0.001) exhibited highly significant fumigant toxicity against *T. castaneum* adults compared to untreated controls and the chlorpyrifos treated group. Carvacrol demonstrated significantly greater fumigant efficacy than *C. capitatus* essential oil. The LC_50_ values for *C. capitatus* essential oil and carvacrol were 168.5 mg/L and 106.5 mg/L, respectively (Table 3).

### 2.4. Effects of Coridothymus capitatus and Carvacrol on F1 Progeny Production of T. castaneum (30–45 Days)

Both *C. capitatus* essential oil and carvacrol showed potent insecticidal activity against adult Tribolium castaneum, with 24 h LC_50_ values of 0.53 mg/g for both compounds. The LC_90_ value was also 0.53 mg/g for *C. capitatus* essential oil, while for carvacrol, it was slightly higher at 1.17 mg/g (Table 4). Both essential oils significantly inhibited progeny production compared to control treatments (*p* < 0.001, F = 24,373.3 for *C. capitatus* and F = 41,339.3 for carvacrol). The complete absence of F1 adults at the LC_90_ level indicated the sterilizing effects of both compounds. Even at the LC_50_, *C. capitatus* and carvacrol demonstrated strong inhibitory effects on progeny production, reducing it by 96% and 98%, respectively (Table 5).

### 2.5. Antifeedant Effects (Grains Weight Loss)

Both essential oils exhibited a highly significant antifeedant effect (*p* < 0.001; F = 514.7) compared to the untreated or chlorpyrifos-treated control insects. When dried wheat grains were treated with LC_90_ concentrations of *C. capitatus* and carvacrol, the weight loss decreased from 29.02% to 10.4% and 5.6%, respectively (Table 6). Carvacrol demonstrated a greater feeding deterrent index (FDI%) than *C. capitatus*, with an FDI% of 80.70% (Table 6).

### 2.6. Residual Toxicity of Carvacrol on Wheat Grains against Adult T. castaneum

**Figure 2:**
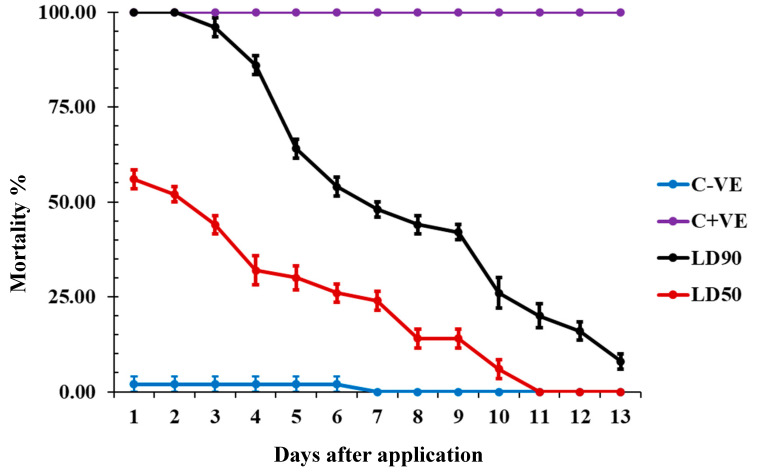
The mortality % recorded from residual toxicity effect of carvacrol against *T. castaneum* adults, where (C − ve) refers to control untreated insects and (C + ve) is control treated with chlorpyrifos.

The LD_90_ and LD_50_ of carvacrol had distinct effects on insect mortality over time. The LD90 caused a significant increase in total mortality for the first two days, followed by a sharp reduction in mortality, eventually reaching only 10% after 12 days. In contrast, the LD_50_ showed a decrease in mortality beginning on the fourth day, with a complete loss of toxicity by the twelfth day after application. This indicates that while higher doses of carvacrol maintain strong initial efficacy, the toxic effects diminish over time, particularly at lower doses. (Figure 2).

## 3. Discussion

The fundamental requirements of any grain storage method or structure are to secure the grains from insects and rodents and to prevent spoilage caused by the activities of micro-organisms [16]. Chemical control methods, such as those employing pyrethroids and organophosphates, offer rapid, cost-effective, and efficient management of stored product pests. However, they also present significant drawbacks, including environmental damage, product contamination, human and animal health risks, pesticide residues, and the development of pest resistance [17]. Consequently, the pursuit of alternative, non-toxic grain protectants has become a paramount objective. This research focused on assessing the insecticidal potential of *C. capitatus* and carvacrol as promising candidates for the control of the red flour beetle, *T. castaneum*, a notorious pest of stored grains.

The present data reveled that *C. capitatus* exhibited pronounced fumigant toxicity, repellent, and antifeedant properties against the red flour beetle, *T. castaneum*. Furthermore, a significant reduction in the beetle’s progeny (F1 generation) was observed, indicating a pronounced impact on reproductive capacity. Previous studies have established the fumigant toxicity of Lamiaceae-derived essential oils against stored product beetles [18]. The present study represents the first investigation into the fumigant toxicity of *C. capitatus* against *T. castaneum*. The current GC-MC analysis revealed the present of several components of *C. capitatus*, e.g., carvacrol, thymol, p-cymene, linalool, and caryophyllene, which were previously documented to exhibit contact toxicity [18]. For instance, p-cymene has demonstrated contact toxicity against *Sitophilus oryzae* with an LC_50_ of 0.8 mg/cm^2^ [19]. Similarly, thymol has been reported to exhibit contact toxicity against *Sitophilus oryzae* and *Rhyzopertha dominica* with LC_50_ values of 8.8 and 24.07 μg/cm^2^, respectively [20]. Linalool has demonstrated potent contact toxicity against four key stored product pests: *Sitophilus oryzae*, *Sitophilus zeamais*, *Lasioderma serricorne*, and *Tribolium castaneum*. The reported LC_50_ values for these pests are 66.74 μg/cm^2^, 2.45 μL/cm^2^, 27.41 μg/cm^2^, and 45.96 μg/cm^2^, respectively [21,22,23]. These findings underscore the contact toxicity of these bioactive compounds and, consequently, the broader insecticidal properties of *C. capitatus* extract against a range of stored product pests.

In the present study, carvacrol exhibited pronounced fumigant, repellent, and antifeedant effects against *T. castaneum*, significantly suppressing adult populations and progeny production, even at low concentrations. Similarly, carvacrol showed significant toxicity against *Sitophilus oryzae* and *T. castaneum* with LC_50_ values of 17.15 and 21.16 μg/cm^2^, respectively [20]. Remarkably, carvacrol revealed significant toxicity against *Lasioderma serricorne* and *Rhyzopertha dominica* with very low LC_50_ values of 0.012 and 0.019 mg/cm^2^, respectively [24].

The present study demonstrated that both EOs exhibited significant repellent effects against adult *T. castaneum*. While the repellent properties of *C. capitatus* EO have not been previously reported, other *Thymus* species have shown repellent activity against stored product insects. For instance, *Thymus vulgaris* has been shown to strongly repel *Plodia interpunctella* and *Sitophilus zeamais* [25,26,27]. Additionally, *Thymus quinquecostatus* effectively repelled *T. castaneum*, *Lasioderma serricorne*, and *Liposcelis bostrychophila* [28], and *Thymus persicus* EO strongly repelled *T. castaneum* and *Callosobruchus maculatus* [29]. Furthermore, carvacrol, a major component of both EOs, has been shown to repel *Rhyzopertha dominica* and *Lasioderma serricorne* with high repellency rates after three hours of exposure at 12.5 μg/cm^2^ [30].

Significantly, both essential oils revealed antifeedant effect against *T. castaneum*. This pronounced response is likely attributed to the high concentration of carvacrol within the oils. Previous research has established carvacrol’s potent antifeedant properties against a broad spectrum of insect pests, including stored product coleopterans. For instance, a 30% feeding deterrence against *Rhyzopertha dominica* was observed at a maximal concentration of 1 mg/g [20]. Moreover, carvacrol exhibited 50% feeding repellency against *Aedes aegypti* mosquitoes at a dosage of 22.51 μg/cm^2^ [31].

The current study represents the first evaluation of the progeny inhibition effects of both essential oils (EOs) against *T. castaneum*. A significant reduction in *T. castaneum* progeny was observed following exposure to both EOs. Previous research by [32] demonstrated a similar effect of carvacrol on *S. zeamais* progeny, with decreasing progeny production at higher concentrations. The noticeable reduction in offspring is likely attributed to disruptions in adult mating and communication, leading to decreased oviposition and potential mortality during the egg and larval stages [33].

Overall, the essential oil (EO) from *C. capitatus* is highly insecticidal in its various modes of action, which are dependent on its chemical composition. This is notably due to the presence of carvacrol, which has insects’ toxic properties [34]. The current results showed that *C. capitatus*’s toxicity can be explained by a very high concentration of carvacrol, reaching 71%. There are two suggested main mechanisms for the observed insecticidal properties of carvacrol. Firstly, at the central nervous system level, octopaminergic and gamma-aminobutyric acid (GABA) receptors are considered as the main targets for the neurotoxic effects of carvacrol. As a result, this interaction induces significant alterations in the neuromuscular transmission in insects [35]. Secondly, carvacrol inhibits acetylcholinesterase activity [36]. In addition, it has been found out that carvacrol exhibits a significant inhibitory effect on insects’ detoxification system by specifically targeting cytochrome P450s [37]. Importantly, thymol, as one of seven monoterpenes investigated, has been proven to be the most effective inhibitor of food intake within the EO composition of *C. capitatus* [38]. The neurotoxic potential of EOs of *Thymus capitatus* is not limited to its insecticidal activity, but includes other symptoms such as hyperactivity, convulsions, and paralysis. The aforementioned symptoms can result in death because the essential oil affects indigenous physiological mechanisms in the insect [34].

*Thyme* essential oils offer numerous advantages as insecticides, positioning them as a viable alternative to synthetic pesticides as they are eco-friendly. *Thyme* essential oils have a low environmental persistence due to their volatility, reducing the likelihood of harmful residues [39]. Furthermore, essential oils of *thyme* demonstrate enhanced targeted efficacy, as they encompass insecticidal properties that selectively target specific pests, while leaving beneficial organisms unaffected, thus contributing to the preservation of the ecological equilibrium. Also, EOs tend to be less harmful to mammals, making them a safer option for environments where humans and pets are present [40]. In addition, *thyme* essential oils are officially approved by agencies such as the EPA and FDA as safe for inclusion in food products, which further increases their attractiveness as a pest control solution [41]. On the other hand, there are some disadvantages of *thyme* oil, e.g., their effectiveness, which can vary widely based on several factors, including the specific type of oil, its concentration, and the environmental conditions. This variability can make them less trustworthy than conventional insecticides [42]. Moreover, exposing essential oil of *thyme* to the various environmental conditions may encounter different stability issues. This instability can lead to a decrease in their efficacy over time, making them less reliable compared to synthetic insecticides [43].

## 4. Materials and Methods

### 4.1. Chemicals

Acetone (99.9%) and chlorpyrifos (25%) were purchased (KZ Chemical Industries, Giza, Egypt), while both *N*, *N*-diethyl-m-toluamide (DEET, 97%), and purified oil of carvacrol (98%) were purchased form Sigma Aldrich in Cairo, Egypt.

### 4.2. Insect Culture

This colony of *Tribolium castaneum* was maintained in a dark incubator at a relative humidity of 70–80% and a temperature of 28 °C for five years, without exposure to any insecticide. The insects were reared on a mixture of active yeast and wheat flour (10:1, *w*/*w*), with the moisture level of the wheat flour being 12–13% according to [44]. One-week-old, unsexed adult insects were used for the following bioassays.

### 4.3. Coridothymus capitatus Essential Oil Extraction

*Coridothymus capitatus* was collected from the vicinity of Institute of Agriculture nearby Izmir and identified by Dr. Aşkın Akpulat, a plant taxonomist at Cumhuriyet University, Sivas, Turkey. The leaves were separated from the stems and blossoms, dried in the shade for two weeks, and then crushed to pass through a 2 mm mesh. A total of 100 g of the dried, ground leaves were placed in a Clevenger-type distillation apparatus with 2 L of double-distilled water and hydrodistilled for 3 h [45]. The extracted oil was dried over anhydrous sodium sulfate, filtered, and stored at 4 °C until use.

### 4.4. GC-MS Analysis of the Essential Oil

The essential oil (EO) was analyzed using a Trace Ultra gas chromatograph (GC) coupled with a DSQ II mass spectrometer (MS; Thermo Scientific, Waltham, MA, USA). The compounds were isolated on a Thermo-5MS (30 m × 0.25 mm ΙD, film thickness 0.25 μm) capillary column (Thermo Scientific) with a temperature program ranging from 60 to 250 °C at a rate of 3 °C/min and a helium flow rate of 1 mL/min. The injector and MS transfer line temperatures were set at 220 and 250 °C, respectively. Samples were prepared by diluting 1 mg of EO in 1 mL of acetone, and 1 µL of the diluted sample was injected manually in splitless mode. The MS operated in EI mode at 70 eV, with an ion source temperature of 230 °C, and mass spectra were acquired in scan mode for the mass range 40–400. The identification of the compounds was achieved by the comparison of their relative retention indexes and mass spectra with corresponding data reported in the literature and instrument’s databases (Adams Book 07, Nist 98, Xcalibur). A series of n-alkanes (C8–C24) was used to determine the relative retention index (RRI). Relative percentages of the compounds were obtained electronically from area percentage data.

### 4.5. Repellency Assay

This assay was conducted in Petri dishes based on the choice behavior of the insects. Glass Petri dishes (9 cm in diameter) were used, with Whatman filter papers (9 cm in diameter) divided into two halves. A pipette was used to evenly distribute 0.5 mL of each dilution onto one half of the filter paper at concentrations of 0.125, 0.25, 0.5, 1, and 2 mg/cm^2^, while the other half of the filter paper was treated with acetone only. The solvent was allowed to evaporate for 10 min. *C. capitatus* extract and carvacrol were prepared in an acetone solvent at different dilution rates to produce the required solutions. The positive control treatment was prepared in the same manner using DEET (2 mg/cm^2^) [24] on one-half of the filter paper and acetone on the other. After the solvent had evaporated, the Petri dishes were covered and 10 adult *T. castaneum* were placed within them, between the two filter paper halves. The bioassay was conducted at room temperature (26 ± 2 °C) and in daylight (10 am) with five replicates of each dilution [46]. After 3 h, the total numbers of *T. castaneum* on the treated (Nt) and untreated halves (Nc) were counted, recorded, and the repellency percentages (PR) were calculated using the following equation [47]: PR = [(Nc − Nt)/(Nc + Nt)] × 100.

### 4.6. Fumigant Toxicity Assay

A 2 cm diameter Filter sheets was placed to cover the inner surfaces of a 20 mL glass vials. With a micropipette, 5 μL/L of the tested concentrations was applied to each piece of paper as follows: 6.25, 12.5, 25, 50, and 100 μL/L of *C. capitatus* extract and carvacrol. Five replications were used for every concentration. A positive control using chlorpyrifos (5 μL/L) and an acetone-treated piece of paper was utilized as a negative control. Following the placement of each vial with its 10 insects, the tops were tightly secured. The vials were maintained under controlled conditions at 30 ± 1 °C and 60–65% relative humidity for 24 h [48]. Insect mortality was determined, and insects were considered dead if there was no movement of legs or antennae after 15 s of stimulation with an entomological pin. The mortality rate was determined using [49] the following correction:

(1) $$\%\;\mathrm{Mortality}=\left[\frac{\%Mt-\%Mc}{100-\%Mc}\right]\times 100$$

where (M_t_) is mortality on treatment and (M_c_) is mortality on control.

### 4.7. Effects of C. capitatus and Carvacrol on F1 Progeny Production of T. castaneum (30–45 Days)

To assess the impact of *C. capitatus* and carvacrol on the progeny production of *Tribolium castaneum*, a modified method of [50] was followed. Wheat grains were treated with *Coridothymus capitatus* and carvacrol at LC_50_ and LC_90_ concentrations and dissolved in acetone. A control group was treated with acetone alone. Ten adult beetles were introduced into plastic cups containing 10 g of treated wheat. The beetles were allowed to oviposit for seven days. After removing the adults, the cups were incubated until the F1 generation emerged (days 30–45). The number of adult F1 beetles was counted in each cup. The inhibition rate of progeny was calculated using the formula provided by [51]:

(2) $$IR\%=\frac{C_{n}-T_{n}}{C_{n}}\times 100$$

where C_n_ and T_n_ refers to number of Fl progeny on control and treated cups, respectively.

### 4.8. Antifeedant Effects (Grains Weight Loss) of C. capitatus and Carvacrol

Dried wheat grains weighing 10 g were divided into five replicates and placed in plastic cups. Each cup was then treated with an LC_90_ concentration of *C. capitatus* extract and carvacrol, with acetone used as a control. The cups were covered with muslin cloth and incubated under controlled conditions for 30 days. The percentage weight loss of the grains was calculated using the following formula: Weight loss% = (W1 − W2)/W1 × 100. This formula compares the grain’s initial weight before the experiment (W1) to its weight after 30 days of the experiment (W2). The antifeeding effect of the essential oils was assessed by measuring the weight loss in the grains and calculating the feeding deterrent index (FDI%). The FDI% was calculated using the following equation: FDI% = (C − T)/C × 100, where C = weight loss in the control untreated grains and T = weight loss in the treated grains [51].

### 4.9. Residual Toxicity of Carvacrol on Wheat Grains against Adult T. castaneum

The long-term pesticide residual impact of the investigated essential oils was evaluated using a modified method based on [52]. Thirty grams of wheat grains were mixed with carvacrol at the LC_50_ and LC_90_ levels in a glass jar measuring 25 cm in diameter and 20 cm in height. To ensure even oil distribution throughout the grains, the mixture was thoroughly shaken and then allowed to sit for an hour to let the solvent evaporate. A total of 10 pairs of adult *T. castaneum* from the stock culture were introduced to 10 g of the treated grains, which had been transferred to clean plastic cups. A parallel control treatment using untreated grains was also conducted. Three replicates of the experiment were performed [52]. Ten adult insects were released every twenty-four hours until minimal mortality of less than 10% was observed [53].

### 4.10. Statistical Analysis

Statistical analyses were conducted using IBM SPSS Statistics version 26. One-way ANOVA followed by Tukey’s post hoc test was employed to identify significant differences among treatments at a significance level of *p* < 0.05. Probit analysis, as described by [54], was used to calculate lethal concentrations (LC_50_ and LC_90_) and their respective 95% confidence intervals. Data are presented as mean ± standard error.

## 5. Conclusions

The present observation confirmed that both *C. capitatus* essential oil and carvacrol exhibited substantial insecticidal efficacy against *Tribolium castaneum*, demonstrating superior performance in repellency, feeding deterrence, progeny inhibition, and mortality rates exceeding 70%. These findings underscore the potential of these natural compounds as a promising, ecofriendly strategy for managing stored product insect pests. The appropriateness of their safety profile and approval by the European Food Safety Authority (EFSA) for use in animal feed applications further boosts them in terms of sustainable grain protection.

## Figures and Tables

**Table 1:** Chemical composition of the *Coridothymus capitatus* essential oil.

K.I.Experimental	Compounds	%Area
926	α-Thujene	0.49
936	α-Pinene	0.54
956	Camphene	0.25
980	1-Octen-3-ol	0.13
989	Myrcene	1.11
1007	α-Phellandrene	0.03
1018	α-Terpinene	0.88
1026	p-Cymene	7.69
1060	γ-Terpinene	8.3
1074	cis-Sabinene hydrate	0.3
1088	Terpinolene	0.02
1100	Linalool	0.49
1174	Borneol	0.95
1181	Terpinen-4-ol	0.81
1195	α-Terpineol	0.03
1197	cis-Dihydro carvone	0.02
1290	Thymol	5.02
1297	Carvacrol	71.14
1366	Carvacrol acetate	0.01
1418	β-Caryophyllene	1.48
1456	α-Humulene	0.06
		99.75
traces: <0.1%		

**Table 2:** Repellent effect of *C. capitatus* essential oil and carvacrol against adults of *T. castaneum* adults (n = 5).

Conc. (mg/cm^2^)	Repellency Percentages (PR) ± SEM	
	*Coridothymus capitatus*	Carvacrol
DEET Control	100 ± 0.00	100 ± 0.00
2	72.0 ± 8.0 *	92.0 ± 4.9
1	48.0 ± 4.9 ***	72.0 ± 4.9 ***
0.5	24.0 ± 4.0 ***	44.0 ± 4.0 ***
0.25	12.0 ± 4.9 ***	28.0 ± 4.9 ***
0.125	12.0 ± 4.9 ***	32.0 ± 4.9 ***
PR 50%	1.05 (0.792–1.54) ^a^	0.42 (0.32–0.54) ^a^
Slope ± SE	1.13 ± 0.03	1.27 ± 0.04
Regression equation	y = −0.09 + 1.13x	y = 0.42 + 1.27x
R^2^	0.72	0.69

R^2^ = the coefficient of determination. ^a^ = 95% lower and upper confidence limits. Data expressed as mean repellency percentages ± SEM. Statistical differences from the respective control treated with *N*, *N*-diethyl-m-toluamide (DEET) are indicated as * (*p* ≤ 0.05), *** (*p* ≤ 0.001).

**Table 3:** Fumigant toxicity effect of *Coridothymus capitatus* essential oil and carvacrol against *T. castaneum* adults (n = 5).

Concentration (mg/L)	Mortality Percent ± SEM	
	*C. capitatus*	Carvacrol
Control untreated (acetone)	0 ± 00	0 ± 00
Control treated with chlorpyrifos 25% (10 mg/L)	100 ± 00 ***	100 ± 00 ***
100	36.00 ± 2.45 ***^###^	50.0 ± 3.16 ***^###^
50	20.00 ± 3.16 ***^###^	32.0 ± 3.74 ***^###^
25	14.00 ± 2.45 ***^###^	22.0 ± 3.74 ***^###^
12.5	6.00 ± 2.45 ^###^	14.0 ± 2.45 ^###^
6.25	0 ± 0.00 ^###^	6.0 ± 2.45 ^###^
LD50	168.47 (117.1–304.1) ^a^	106.5 (77.09–174.02) ^a^
Slope ± SE	1.27 ± 0.34	1.24 ± 0.25
Regression equation	y = −2.93 + 1.27x	y = −2.5 + 1.24x
R^2^	0.98	0.99

R^2^ = the coefficient of determination. ^a^ = 95% lower and upper confidence limits. Data are mean mortality percent ± SEM. Statistical differences from the respective control -ve are indicted as *** (*p* ≤ 0.001), from control treated with chlorpyrifos (C + ve) as ^###^ (*p* ≤ 0.01).

**Table 4:** LC_50_ and LC_90_ of the *Coridothymus capitatus* and carvacrol against *Tribolium castaneum* in wheat grains after 24 h.

Concentration (mg/g)	Carvacrol	*C. capitatus*
LD_50_	0.53 (0.42–0.61) ^a^	0.72 (0.59–0.84) ^a^
LD_90_	1.17 (1.03–1.41) ^a^	2.48 (2.02–3.39) ^a^
Slope ± SE	2.86 ± 0.5	2.21 ± 0.04
Regression equation	y = 0.89 + 2.86x	y = 0.33 + 2.21x
R^2^	0.81	0.89

R^2^ = the coefficient of determination. ^a^ = 95% lower and upper confidence limits.

**Table 5:** The inhibition rates of progeny by the effect of *C. capitatus* and carvacrol against *T. castaneum* adults (n = 5).

Substances	Concentration (mg/g)	Inhibition Rates	Mean Number of Emerged Adults ± SEM
Control untreated		0 ± 0.00	28.6 ± 0.4
Control treated with chlorpyrifos 25%	(0.01 mg/g)	100 ± 0.00 ***	0 ± 0.00 ***
*Coridothymus capitatus*	LD_90_	100 ± 0.00 ***	0± 0.00 ***
	LD_50_	96.00 ± 0.6 ***	4 ± 0.63 ***
Carvacrol	LD_90_	100 ± 0.00 ***	0 ± 0.00 ***
	LD_50_	98.80 ± 0.49 ***	1.2 ± 0.49 ***

Statistical differences from the respective control are indicated as *** (*p* ≤ 0.001).

**Table 6:** Antifeedant activity and feeding deterrent index of *C. capitatus* and carvacrol at LD_90_ against adult *T. castaneum* adults (n = 5).

Treatment Group	Grain Weight (g) Pre-Treatment	Grain Weight (g) Post- Treatment	Seed Weight Loss %	FDI %
Control untreated	10.0 ± 0.00	7.10 ± 0.06	29.02	0.00
Control treated with chlorpyrifos 25% (0.01 mg/g)	10.0 ± 0.00	10.0 ± 0.00	0.00	100.00
*C. capitatus* LD_90_	10.0 ± 0.00	8.96 ± 0.05	10.4	64.16
Carvacrol LD_90_	10.0 ± 0.00	9.44 ± 0.08	5.6	80.70
	*p* value (*p*< 0.001)		F = 514.7	

FDI% = feeding deterrent index.

## Data Availability

All related data to this work are available in this manuscript.
